# The Integration of the Image Sensor with a 3-DOF Pneumatic Parallel Manipulator

**DOI:** 10.3390/s16071026

**Published:** 2016-07-01

**Authors:** Hao-Ting Lin, Mao-Hsiung Chiang

**Affiliations:** 1 Department of Mechanical and Computer-Aided Engineering, Feng Chia University, No. 100, Wenhwa Road, Seatwen, Taichung 40724, Taiwan; haotlin@fcu.edu.tw; 2 Department of Engineering Science and Ocean Engineering, National Taiwan University, No. 1, Sec. 4, Roosevelt Rd., Taipei 106, Taiwan

**Keywords:** image recognition, parallel manipulator, pneumatic servo system, speed up robust feature algorithm, random sample and consensus algorithm, hand gesture recognition

## Abstract

The study aims to integrate the image sensor for a three-axial pneumatic parallel manipulator which can pick and place objects automatically by the feature information of the image processed through the SURF algorithm. The SURF algorithm is adopted for defining and matching the features of a target object and an object database. In order to accurately mark the center of target and strengthen the feature matching results, the random sample and consensus method (RANSAC) is utilized. The ASUS Xtion Pro Live depth camera which can directly estimate the 3-D location of the target point is used in this study. A set of coordinate estimation calibrations is developed for enhancing the accuracy of target location estimation. This study also presents hand gesture recognition exploiting skin detection and noise elimination to determine the active finger count for input signals of the parallel manipulator. The end-effector of the parallel manipulator can be manipulated to the desired poses according to the measured finger count. Finally, the proposed methods are successfully to achieve the feature recognition and pick and place of the target object.

## 1. Introduction

In recent years, more and more countries have developed various kinds of robots to render human’s lives much more convenient. Abundant literature on robots has been published and used for several decades. For instances, robots are widely adopted in automobile, mechanical, aerospace, medical applications. In this research, the industrial manipulator, the parallel manipulator, will be presented and implemented. This kind of manipulator possesses a high ratio of rigidity to weight, high stiffness, high accuracy and high response, so parallel manipulators have become more popular in diverse industries to handle complex and harsh tasks. In most robot application research, the interaction in the workspace between robots and workpieces is a critical issue. Especially, position mismatch may cause a failure of the functioning. In recent years, visual systems have become the most outstanding method applied in the robot-vision system. To achieve such vision-guided system, the robot should be able to recognize the target object and determine the pose of the object so as to grasp it. In 1988, the Harris corner detector was suggested for the feature detector [1]. Furthermore, the robot needs to modify its motion trajectory according to the target object’s poses. In 2011, the 3D parallel mechanism robot with a stereo vision measurement system was presented by Chiang et al. [2,3]. The stereo vision measurement system is a noncontact measuring strategy using two parallel CCDs to capture the 3D poses of the end-effector instead of the contact displacement sensors. The system can determine the location of the end-effector in the three-dimensional Cartesian coordinate system. In 2016, the 3D visual data-driven spatiotemporal deformations for non-rigid object gasping using robot hands was introduced by Mateo et al. [4]. The experiments show that the proposed method can grasp several objects in various configurations.

Recently, ASUS (Taipei, Taiwan) launched the ASUS Xtion Pro Live camera, a 3D camera system which consists of both an RGB sensor and a depth sensor for capturing color images and per-pixel depth information simultaneously. This device can largely resolve the major problem which is using the images from a 2D camera system to reconstruct the 3D object information in the vision-guided robot. Furthermore, Human-Robot Interaction (HRI) plays a critical role in accomplishing interactive tasks between human and robots. Many researches focus on kinematics, communication, computer vision and control systems, making HRI an inherently interdisciplinary endeavor. Gesture-based interfaces hold the promise of making HRI more natural and efficient [5,6].

This paper combines the depth camera and the 3-DOF pneumatic parallel manipulator, instead of the stereo vision system which is more expense and time consuming, for estimating the 3D location of objects. In addition, the gesture is used as a signal for the manipulator to grasp the desired bodies. The HRI renders the entire system friendly. In a nutshell, a 3-DOF pneumatic parallel manipulator with an image sensor system is successfully developed and implemented.

## 2. System Overview

### 2.1. Mechanism

**Figure 1:**
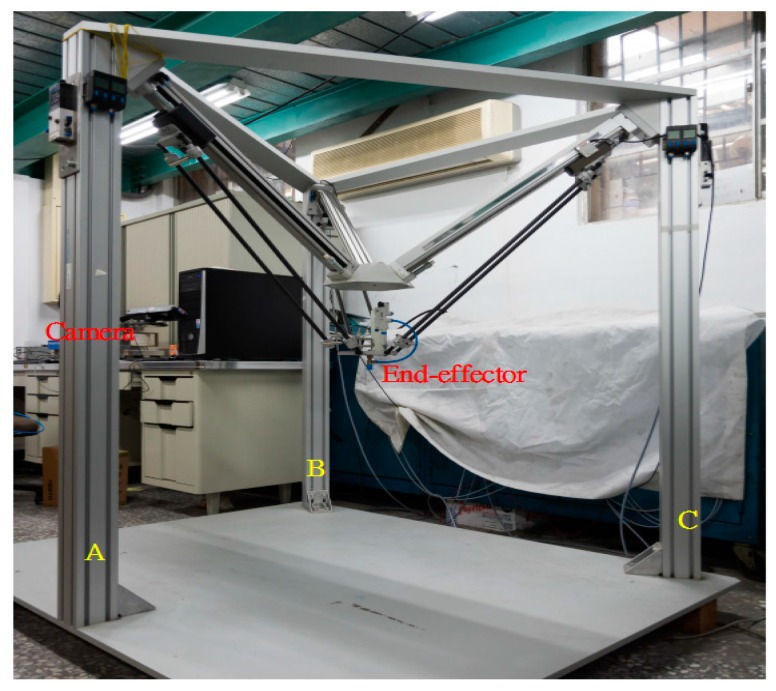
A 3-DOF pneumatic parallel manipulator.

The proposed parallel manipulator is a 3-DOF parallel manipulator by the pneumatic servo system. Figure 1 shows a photograph of device. Three limbs driven by rod-less pneumatic actuators are assembled and connected to the fixed base in the way that the geometric structure of the manipulator is in an inverted pyramidal shape. The three sliders are translated along the linear guide-ways by three 1-DOF prismatic joints driven by the pneumatic rod-less cylinders. The moving platform is linked to each slider by 3-DOF spherical joints. Mobility analysis by the Grübler-Kutzbach formula verifies that the proposed manipulator is a 3-DOF mechanism with its moving platform possessing only translational motion. Furthermore, the 3D camera system, an ASUS Xtion Pro Live depth camera, is set up on the A axis of the parallel manipulator for non-contact measurements. The camera system can directly capture the 3D information of the object by color images and the depth position of each pixel [7].

### 2.2. Test Rig Layout

**Figure 2:**
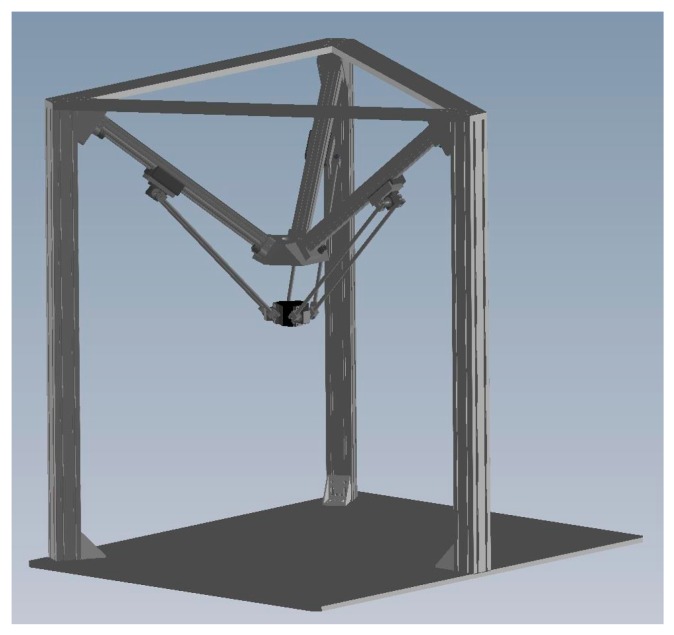
The structure of the 3-DOF parallel manipulator.

**Figure 3:**
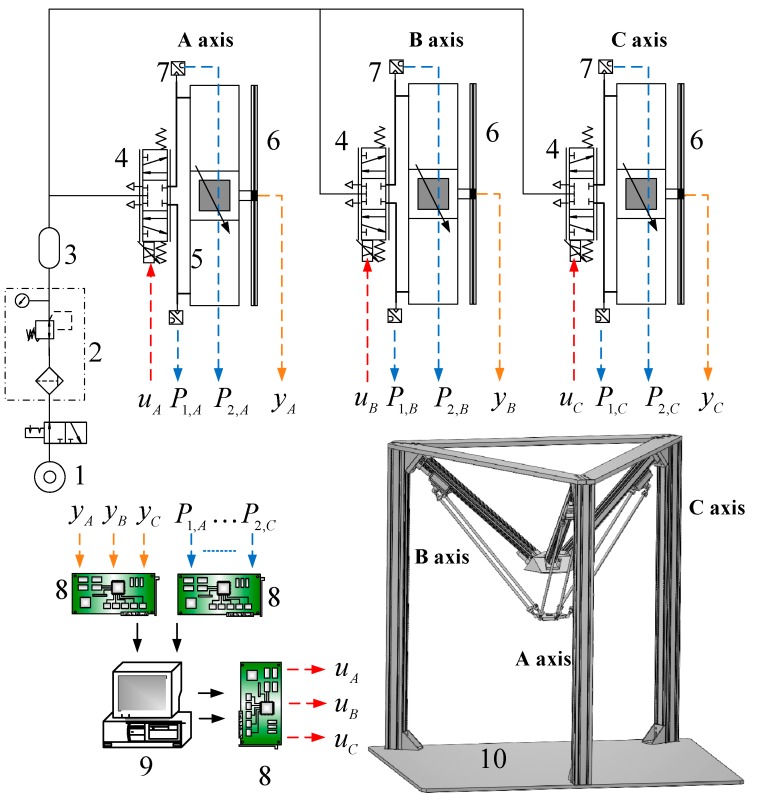
Test rig layout of the 3-DOF pneumatic parallel manipulator. 1: Air Pump; 2: Air Preparation Unit; 3: Air Reservoir; 4: proportional directional control valve; 5: pneumatic rod-less cylinder; 6: optical linear encoder; 7: pressure sensor; 8: interface card; 9: PC-based controller; 10: 3-DOF pneumatic parallel manipulator.

The structure of the 3-DOF parallel manipulator which includes the geometric structure and the linkage configuration is illustrated in Figure 2. The test rig layout of the 3-DOF pneumatic parallel manipulator developed in this research is shown in Figure 3. The upper Figure 3 indicates the pneumatic servo system for driving the 3-DOF parallel manipulator. The overall pneumatic servo system mainly contains an air pump, three proportional directional flow control valve (model MPYE-M5, Festo, Esslingen am Neckar, Germany) and three pneumatic rodless cylinders (Festo model DGC-25-500). In addition, for gauging the real position information of each slider, the position sensor with 1 μm resolution is utilized and attached to each pneumatic actuator. Two pressure sensors are also installed on each cylinder to measure the pressures of the two cylinder chambers. 

Both the measured position signals (*y_A_, y_B_, y_C_*) and chamber pressure signals (*P*_1,*A*_, *P*_2*,A*_,…*P*_2,*C*_) are back to a PC-based controller through the counters and A/D converters on the DAQ card. The input command voltage for the servo valve is given from the analogue output ports on the DAQ card via the D/A converters. The control hardware system which adopts the Matlab Simulink and Mathworks can easily design and realize in the real-time system. The overall algorithms are built up using Matlab Simulink through embedded Matlab function blocks. Furthermore, in Mathworks the Real Time Windows Target (RTWT) can automatically translate the Simulink model into C codes. Also, the control system is implemented on a Windows-based personal computer with 1 kHz of sampling frequency to implement the real-time control system.

## 3. Object Recognition

In this paper, a SURF algorithm, a fast detector and descriptor, is utilized and developed to compute and detect in reducing the feature complexity and enhancing the robustness.

### 3.1. Interest Point Detection

The points of interest are detected by the Hessian-matrix approximation technique. The “Fast-Hessian” detector proposed by Viola and Jones can largely reduce the computational time to detect the object rapidly [8]. Also, Simard proposed a fast convolution algorithm for integral images into the general framework of boxlets [9].

#### 3.1.1. Integral Image

At *X* = (*x,y*)*^T^*, the integral image is the sum of all pixels in a rectangular area set up by the origin and *X*. The integral images are easily and quickly to compute in the box type convolution filters. Choosing positions in the scale, a constant number of entries in a single integral image should be focused on. Also, the image size will mainly dominate the calculation time.

#### 3.1.2. Hessian Matrix Based Interest Points

The advantage of the SURF feature detector with the Hessian matrix is its accuracy performance. The Hessian matrix *H*(*X,σ*) in a location *X* = (*x,y*)*^T^* of an image I with the scale σ can be expressed as:

(1) $$H\left(X,\sigma \right)=\left[\begin{array}{cc} G_{xx}(X,\sigma ) & G_{xy}(X,\sigma )\\ G_{xy}(X,\sigma ) & G_{yy}(X,\sigma ) \end{array}\right]$$

where *G_xx_*(*X*,*σ*), *G_xy_*(*X*,*σ*) and *G_yy_*(*X*,*σ*), the convolution of the Gaussian second order derivative with the image *I* in a location *X*, are $\frac{\partial^{2}}{\partial x^{2}}g\left(\sigma \right),\;\frac{\partial^{2}}{\partial x\partial y}\;g\left(\sigma \right),\;and\;\frac{\partial^{2}}{\partial y^{2}}g\left(\sigma \right).$ When *G_xx_*(*X,σ*) and *G_yy_*(*X,σ*) are positive, and *G_xy_*(*X,σ*) is negative, the maximum will occur. In addition, *D_xx_*, *D_yy_* and *D_xy_* are 9 × 9 box filters. The determinant of approximation is expressed as:

(2) $$\det(H_{approx})≅D_{xx}D_{yy}-{\left(0.9D_{xy}\right)}^{2}$$

where 0.9 is the relative weight of the filter responses for balancing the Gaussian kernel errors.

#### 3.1.3. Scale Space Representation

**Figure 4:**
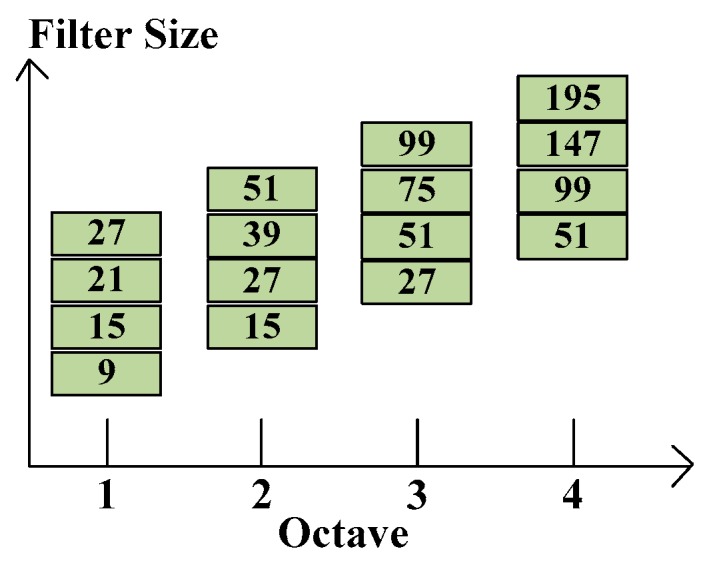
Various side lengths for four different octaves.

Feature of interest points are located in various scales and an image pyramid can realize scale spaces. Lowe [10] proposed that cutting pyramid layers can find the edges and blobs of images. The scale space can separate into octaves which denote filter response maps from convolving the same image in different size filter. Each octave has a constant ratio for scale levels, so the layer can be determined by calculating determinant of approximated Hessian matrix of the same input image in growing size filter. Figure 4 shows the relation between each octave and various filter sizes. Note that the octaves are overlapping in order to cover all possible scales seamlessly. The layer denotes a series of filter response maps obtained by calculating determinant of approximated Hessian matrix of the same input image with a filter of increasing size in each octave.

If the intensity of the central pixel (marked with a cross) is higher than the intensities of its surrounding pixels, including eight pixels around feature point and nine pixels in first and third layer (27 pixels totally), it is considered as a local maximum [11].

#### 3.1.4. Point of Interest Localization

Finding the point of interest, the blob responses of the same neighborhood (denoted as *H*) be taken in each dimension around the detected maximum as described above. Then, locating the maxima to sub-pixel/ sub-scale accuracy through a 3D quadratic to the scale space blob-response map.

(3) $$H(X)=H+{\left(\frac{\partial H}{\partial X}\right)}^{T}X+\frac{1}{2}X^{T}\frac{\partial^{2}H}{\partial X^{2}}X$$

where *X* = (*x,y,σ*)*^T^* are the coordinates of the scale-space. *H*(*X*) means the blob-response at the location *X*. The quadratic coefficients can be approximated by a 2nd order Taylor series approximation of the neighboring samples:

(4) $$\hat{X}=\left[\begin{array}{l} \hat{x}\\ \hat{y}\\ \hat{\sigma } \end{array}\right]-{\left(\frac{\partial^{2}H}{\partial X^{2}}\right)}^{-1}\frac{\partial H}{\partial X}$$

Substituting the above expression into Equation (3):

(5) $$H(\hat{X})=H+\frac{1}{2}[\begin{array}{ccc} \frac{\partial H}{\partial x} & \frac{\partial H}{\partial y} & \frac{\partial H}{\partial \sigma } \end{array}]\hat{X}$$

$│H\left(\hat{X}\right)│\geq 0.03$ we regard it as high contrast point and update best interest points $X_{best}=X+\hat{X}.$ However, $│H\left(\hat{X}\right)│>0.03$ has to be discarded because of low contrast.

### 3.2. Feature Points Matching

Matching interest points of two images will occur in the smallest Euclidean distance:

(6) $$d(P_{i},Q_{i})=\min{\left({\sum_{i=1}^{64}\left\|P_{i}-Q_{i}\right\|}^{2}\right)}^{\frac{1}{2}}$$

$P_{i}$ and $Q_{i}$ are two feature points in two images. However, there are still some mismatches in two images. For image transformation, mapping each $x_{i}$ to $x_{i}^{'}$, the homography matrix *H* can be written in Equation (7):

(7) $$X^{\prime }=\left(\begin{array}{l} x_{1}^{\prime }\\ x_{2}^{\prime }\\ x_{3}^{\prime } \end{array}\right)=\left[\begin{array}{ccc} h_{11} & h_{12} & h_{13}\\ h_{21} & h_{22} & h_{23}\\ h_{31} & h_{32} & h_{33} \end{array}\right]\left(\begin{array}{l} x_{1}\\ x_{2}\\ x_{3} \end{array}\right)=HX$$

According to [12] the RANSAC algorithm is the robust estimation technique to attain the estimated parameters for homographies. The putative correspondences and the inlier correspondences can be adopted in the RANSAC algorithm [13]. Four correspondences are to define a homography and the sample numbers are based on the outliers from each consensus state. The detail process can be described as follows:

1. Randomly chose four matching correspondences.
2. Check whether these points are collinear, if so, redo the above step.
3. Compute the homography $H_{curr}$ by normalized DLT from the four points pair.
4. For each putative correspondence, calculate Euclidean distance between two points $d_{i}\;=\;d\left(x_{i}^{'},H_{curr}x_{i}\right)\;+\;d\left(x_{i},H_{curr}^{-1}x_{i}^{'}\right)$ by the above $H_{curr}$.
5. Count the number of inliers m which has the distance $d_{i}\;<\;T$ (threshold).
6. Repeat above steps until sufficient number of inlier pairs are counted.
7. Update best $H=\;\;H_{curr}$ and record all the inliers.
8. Using normalized DLT algorithm to recompute the homography from all consistent correspondences (inliers).

After applying the RANSAC algorithm, we can see that this efficiently eliminates those inaccurate correspondences. Because homography has the property of being scale- and rotation-invariant, we can highlight precisely the targets in the current image plane. Once the correct homography H be calculated, we can find the desired object in complicated backgrounds by averaging four corners of the reference image after applying a homogenous transformation.

## 4. Gesture Recognition

**Figure 5:**
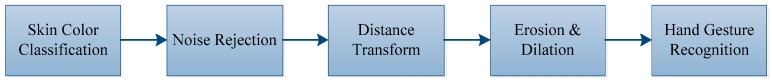
Gesture Recognition Process.

Figure 5 shows the hand gesture recognition process. The gesture can be determined via finger numbers for controlling the manipulator to grasp the specified objects.

### 4.1. Skin Color Classification

Although the RGB model can reduced the large time needed for computer graphics design, it is still hard to execute image processing algorithms due to the fact the RGB color components are extremely correlated. In order to enhance the allowance for image intensity, RGB images can be transformed into a HSI color space, so intensity and chromaticity can be separated. Equation (8) is for RGB image transfer to HSI color space [14]:

(8) $$\begin{array}{l} H=\cos^{-1}\left[\frac{\left[\left(R-G)+(R-B\right)\right]/2}{\sqrt{{\left(R-G\right)}^{2}+(R-B)(G-B)}}\right]\\ S=1-\frac{3}{R+G+B}[\min(R,G,B)]\\ I=\frac{1}{3}(R+G+B)\\ If\;B\;is\;greater\;than\;G,\;then\;H=360^{o}-H \end{array}$$

**Figure 6:**
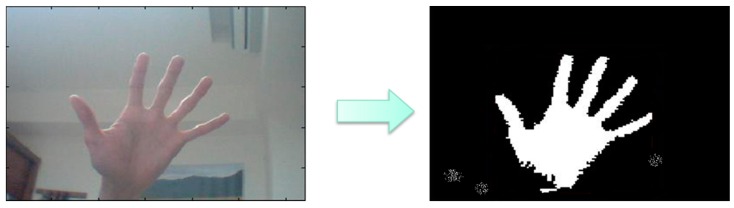
The result of skin color segmentation.

The RGB model of the image from the webcam can be converted to HSI color space because skin color is easily identified. The hue value should be between 0.4 and 0.6 and the saturation value also should be between 0.1 and 0.9. Figure 6 shows the results of skin color segmentation.

(9) 0.4<H<0.6                and            0.1<S<0.9

### 4.2. Noise Rejection

In a general environment situation, we can’t guarantee the image background will be clear. There will be some skin-like objects in the image, which produce unexpected noise. In that case, we use an area condition to filter out noises. First, we calculate the pixel area of each connected component B(i,j) by Equation (10) as follows:

(10) $$Area=\sum_{i=1}^{N}\sum_{j=1}^{M}B(i,j)$$

**Figure 7:**
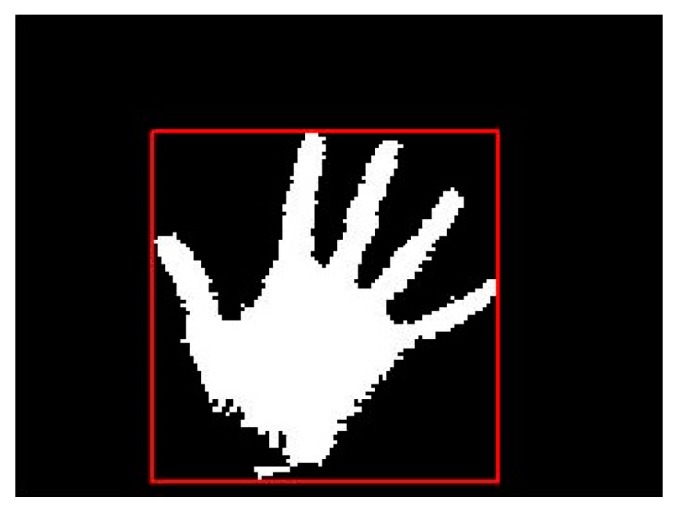
Noises be rejected by the area selection method.

After applying area filter method, the result is shown in Figure 7.

### 4.3. Distance Transform

**Figure 8:**
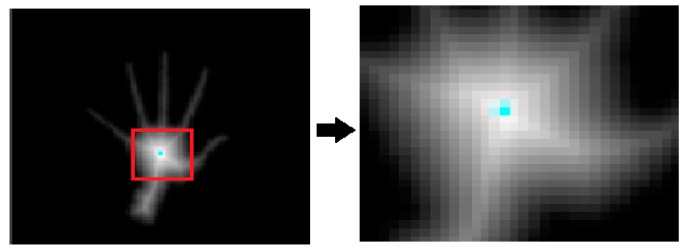
Image showing the hand region after applying distance transform (**left**) and the enlarged view of the region within the red rectangle (**right**).

The distance transform means that the distance from the boundary to a pixel in the hand region increases as the pixel is away from the boundary [15]. Using this distance value, the centroid of the palm region can be calculated. Figure 8 (left) shows the image of the hand after applying the distance transform. The right image of Figure 8 demonstrates the enlarged view of the region within the red rectangle.

The white color in the center is intense and the color fades when the distance increases. The pixels near the boundary have lower values for distance and the pixels away from the boundary have higher values for distance. This middle region which has the highest value for the distance is considered as the centroid.

### 4.4. Morphology

**Figure 9:**
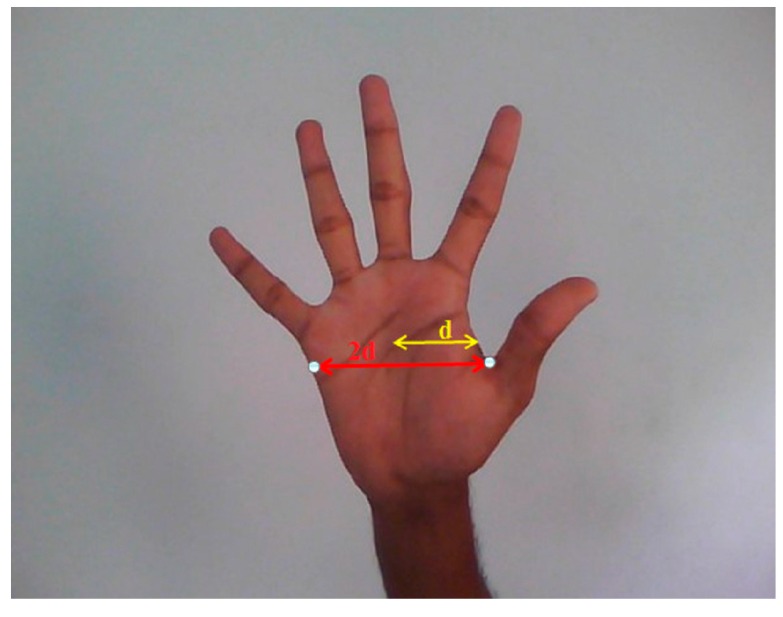
Image of the hand width.

The width of the hand region will be approximately twice the distance from centroid to the nearest boundary pixel as shown in Figure 9. 

The width of each finger is approximately one fourth of the width of the hand. Now a suitable structuring element S that can erode the fingers completely is chosen and erosion is performed on the segmented hand region.

(11) $$R_{p1}=I⊖S$$

**Figure 10:**
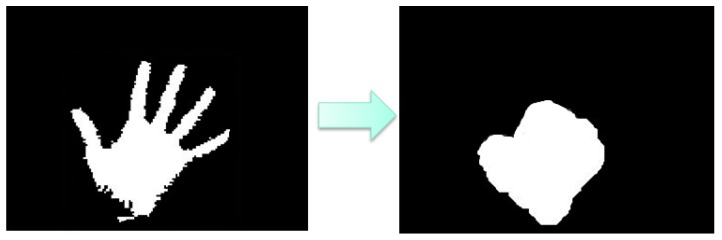
Left image is hand region binary image, the right image is $R_{p2}.$

After erosion only a part of the palm region $R_{p1}$ is left behind and the finger region is completely eroded. Further the palm region which remains after erosion $R_{p1}$ is dilated using the same structuring element and this give the region $R_{p2}$ which is larger than the dilated palm region. The result of $R_{p2}$ is shown in Figure 10:

(12) $$R_{p2}=R_{p1}⊕S$$

**Figure 11:**
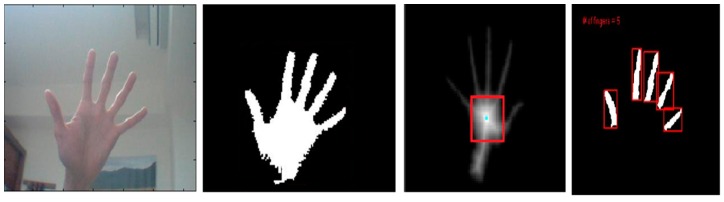
The image processing results.

The dilated palm region $R_{p2}$ is from the original binary image I to the finger area $F_{R}$ . alone as shown in Figure 11.

(13) $$F_{R}=I-R_{p2}$$

The finger numbers represent the gesture is found by the image $F_{R}$.

## 5. 3D Object Localization

After applying the image processing algorithm described in the previous sections, we can recognize desired feature points in RGB color images and depth images. The problem we are dealing with is how to estimate the feature point location in 3D world coordinates (the manipulator end-effector frame).

### 5.1. Calibration of Depth Camera

**Figure 12:**
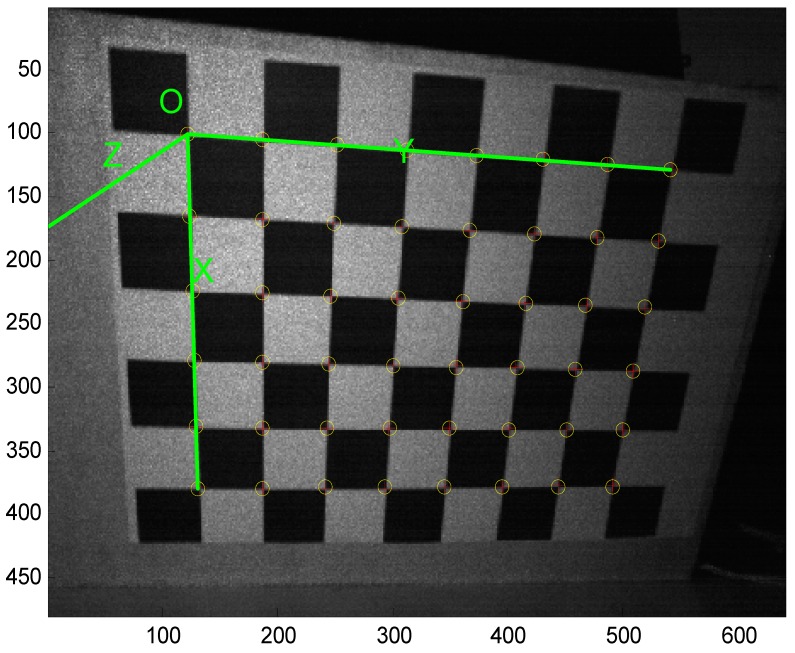
Corner extraction process.

Bouguet adapted the calibration method of Zhang [16] which employs a chessboard to be the calibration pattern. Figure 12 shows the corner extraction process. “+” is for image points and “o” is for re-projected grid points.

After obtaining the depth camera’s image, the intrinsic parameters can be calculated by the camera calibration toolbox. Table 1 illustrates the depth camera’s intrinsic parameters.

### 5.2. Object 3D Location via Depth Camera

The depth camera returns a raw depth data x which has 11 bits resolution, and depth information ranges from 0 to 2047. The depth distance Z can be obtained from the raw depth data converted into depth image by the camera. The following equations show the depth distance as [17]:

(14) $$Z=a_{1}\times exp(-{\left((x-b_{1})/c_{1}\right)}^{2})+a_{2}\times exp(-{\left((x-b_{2})/c_{2}\right)}^{2})$$

where:

$$\begin{array}{ll} a_{1}=3.369\times 10^{4} & a_{2}=6.334\times 10^{18}\\ b_{1}=1338.0 & b_{2}=2.035\times 10^{4}\\ c_{1}=140.4 & c_{2}=3154.0 \end{array}$$

Once the depth distance from the camera and the intrinsic parameters of the camera model are known, we can estimate 3D location of desired feature points in depth images. According to [16], the accuracy of 3D object localization can be determined as follows:

(15) $$\begin{array}{l} X=\frac{Z(u-c_{x})}{f_{x}}\\ Y=\frac{Z(v-c_{y})}{f_{y}} \end{array}$$

where (*X, Y, Z*) is the 3D location of the feature point, (*c_x_*,*c_y_*) is the distance from the optic axis, and (*u*,*v*) is the homogenous pixel coordination.

### 5.3. Hand-Eye Coordinates Calibration

**Figure 13:**
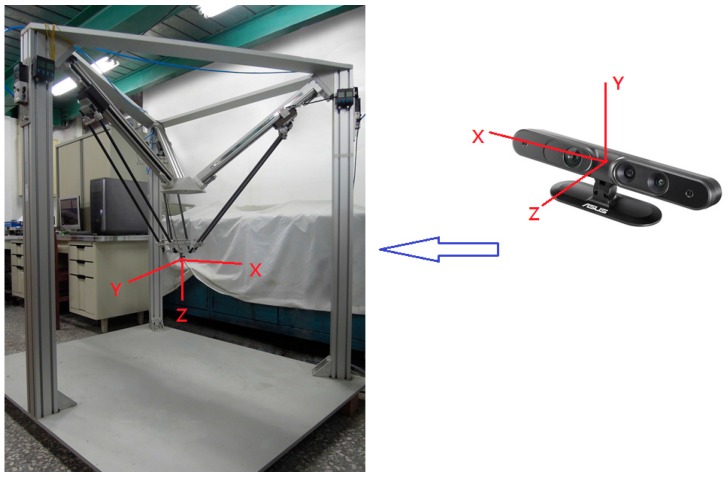
The coordination relationship.

Figure 13 shows the relation coordination between the end-effector and the depth camera. This calibration requires a red color maker as feature point attached to the end-effector.

**T**he transformation between the the Xtion Pro Live depth camera coordinates and the manipulator end-effector reference frame can be written as follows:

(16) $$H_{end-eff}^{cam}P_{cam}=P_{end-eff}$$

where $P_{cam}={\left[x_{c}\;y_{c}\;z_{c}\;1\right]}^{T}$ is a position frame of the maker in the depth camera. Thus, the parameter $P_{end-eff}={\left[X_{end-eff}\;\;Y_{end-eff}\;Z_{end-eff}\;1\right]}^{T}$, is a position of the maker attached on end-effector in the end-effector reference frame. Then, the maker are attached at the center of the gripper. A homogeneous matrix, $H_{end-eff}^{cam}$ includes 12 parameters from the depth camera coordination to the robot end-effector reference frame. Therefore, we can rewrite the Equation (16) as follows:

(17) $$H_{end-eff}^{cam}P_{cam}=\left[\begin{array}{cccc} r_{11} & r_{12} & r_{13} & t_{1}\\ r_{21} & r_{22} & r_{23} & t_{2}\\ r_{31} & r_{32} & r_{33} & t_{3}\\ 0 & 0 & 0 & 1 \end{array}\right]\left[\begin{array}{c} x_{c}\\ y_{c}\\ z_{c}\\ 1 \end{array}\right]=P_{end-eff}=\left[\begin{array}{c} X_{end-eff}\\ Y_{end-eff}\\ Z_{end-eff}\\ 1 \end{array}\right]$$

For solving the twelve unknown parameters, nine rotational operators and three translational operators, ten different end-effector positions will be considered and mapped in Equation (17) in the experiments. Also, the $H_{end-eff}^{cam}$, the fixed relationship between the depth camera coordinates and the end-effector reference frame, is definitely the time invariant matrix, so altering the manipulator to desired poses and using the Xtion Pro Live to extract red feature points on the end-effector, the following transition matrix is described according to least squares method computation:

(18) $$H_{end-eff}^{cam}=\left[\begin{array}{cccc} -0.002 & -0.994 & 0.001 & -29.981\\ 0.584 & -0.000 & 0.811 & 201.185\\ 0.815 & -0.005 & -0.587 & 557.213\\ 0 & 0 & 0 & 1 \end{array}\right]$$

## 6. Experiments

In the previous chapter, the Speed-Up Robust Feature detection with RANSAC algorithm and the finger counting Human-Robot Interaction as well as the coordinate transformation have been analyzed and derived. In this chapter, the SURF object recognition algorithm will be confirmed before finding the desired pokers and estimating their location of each center of pattern in the manipulator reference frame. In next step, we use finger counting HRI to command the manipulator to grasp the selected target. After knowing location of targets and placing locations where we set, the program will automatically generate a 5th order trajectory for the end-effector to pick and place in a three dimensional system. The equation of the 5th order trajectory is as follows:

(19) $$x_{d}\left(t\right)=a_{0}+a_{1}t+a_{2}t^{2}+a_{3}t^{3}+a_{4}t^{4}+a_{5}t^{5}$$

where $a_{0}=x_{d_{0}}$; $a_{1}={\dot{x}}_{d_{0}}$; $a_{2}=\frac{1}{2}{\ddot{x}}_{d_{0}}$; $a_{3}=\frac{1}{2t_{f}^{3}}\left[20x_{d_{f}}-20x_{d_{0}}-\left(8{\dot{x}}_{d_{f}}+12{\dot{x}}_{d_{0}}\right)t_{f}-3\left({\ddot{x}}_{d_{0}}-\;{\ddot{x}}_{d_{f}}\right)t_{f}^{2}\right]$; $a_{4}=\frac{1}{2t_{f}^{4}}\left[30x_{d_{0}}-30x_{d_{f}}+\left(14{\dot{x}}_{d_{f}}+16{\dot{x}}_{d_{0}}\right)t_{f}+3\left({\ddot{x}}_{d_{0}}-2\;{\ddot{x}}_{d_{f}}\right)t_{f}^{2}\right]$; $a_{5}=\frac{1}{2t_{f}^{5}}\left[12x_{d_{f}}-12x_{d_{0}}-\left(6{\dot{x}}_{d_{f}}+16{\dot{x}}_{d_{0}}\right)t_{f}-\left({\ddot{x}}_{d_{0}}-\;{\ddot{x}}_{d_{f}}\right)t_{f}^{2}\right]$.

**Figure 14:**
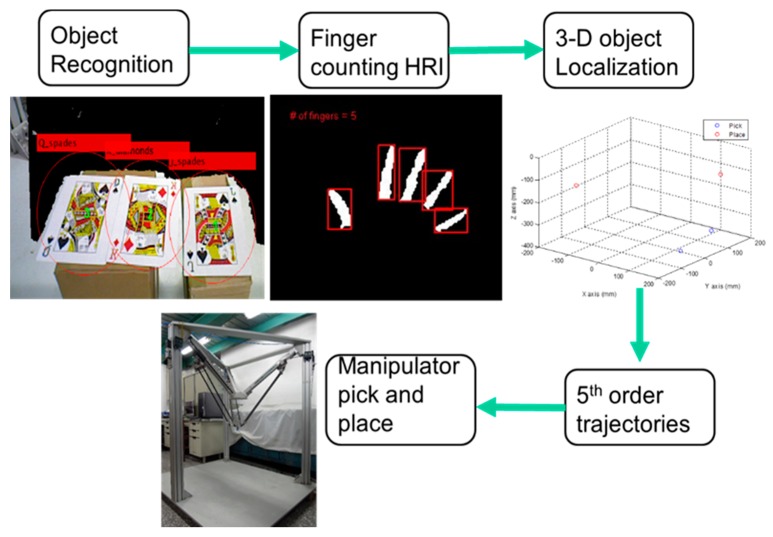
The experiment process.

**Figure 15:**
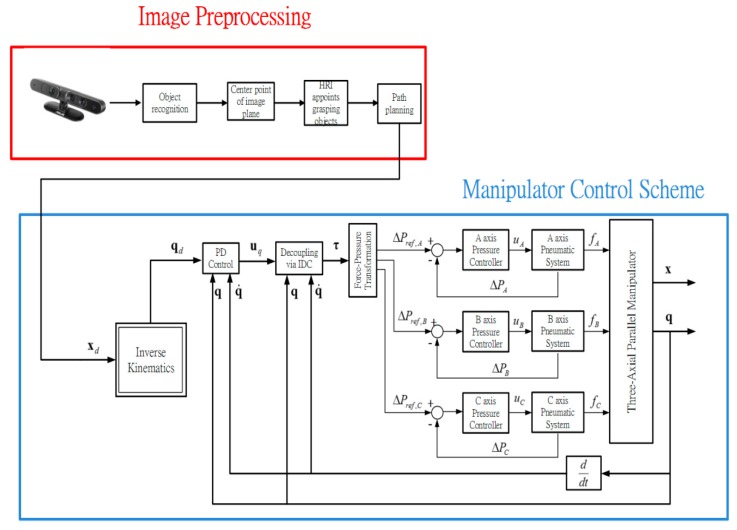
Overall manipulator system control scheme.

$x_{d_{0}},\;{\dot{x}}_{d_{0}}\;and\;{\ddot{x}}_{d_{0}}$ are the position, the velocity and the acceleration at *t* = 0. $x_{d_{f}},\;{\dot{x}}_{d_{f}}\;and\;{\ddot{x}}_{d_{f}}$ are the position, the velocity and the acceleration at *t* = *t_f_* and *t_f_* is the terminal time of the 5th order trajectory. The whole experiment process is illustrated in Figure 14 and the overall manipulator control scheme is illustrated in Figure 15.

**Figure 16:**
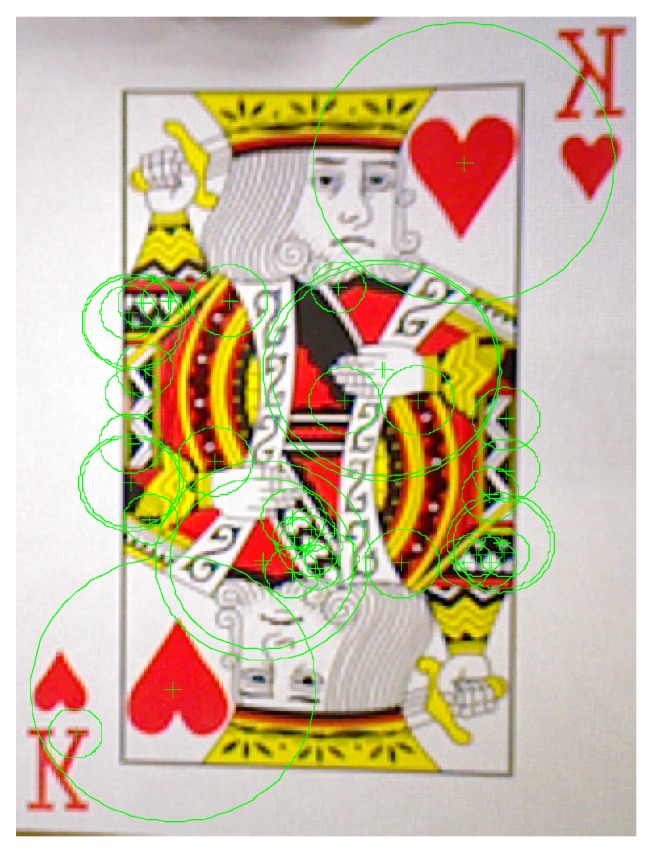
Features of the king of hearts pattern.

**Figure 17:**
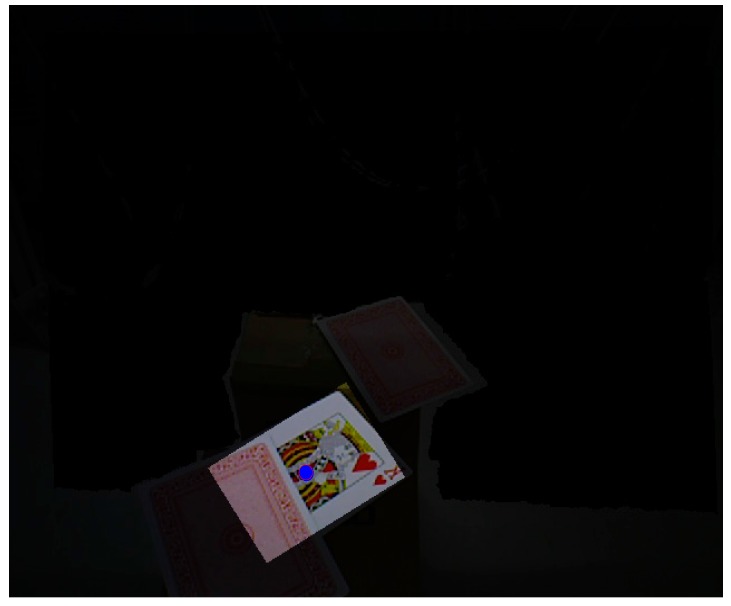
Successful pattern recognition.

**Figure 18:**
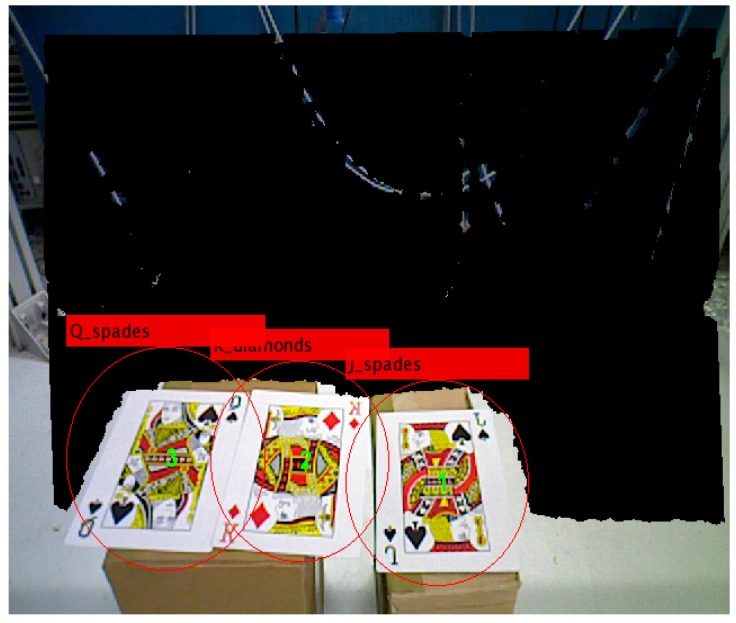
Label numbers on each pattern.

We use poker K, Q and J patterns to construct the database and applied six scale levels in the 3th octave for feature extraction. The king of hearts result is shown in Figure 16. The green crosses denote feature points locations and circles are feature points found in different scale space with 6 s radius. Figure 17 and Figure 18 illustrate the results of the RANSAC algorithm applied to find the inlier correspondences and recognized patterns.

The king of diamonds and the jack of spades are chosen, so the finger counting result must be one and two to select the desire patterns. By using the coordinate transformation, the center points of the poker cards are shown as:

$$\left[\begin{array}{ll} 197.9668 & 189.4207\\ 31.3695 & -120.7430\\ -293.3998 & -323.8801 \end{array}\right]$$

After grasping the target, we need to determine the location to place it. The placment location is as follows:

$$\left[\begin{array}{ll} 100 & -150\\ 200 & -100\\ -150 & -150 \end{array}\right]$$

**Figure 19:**
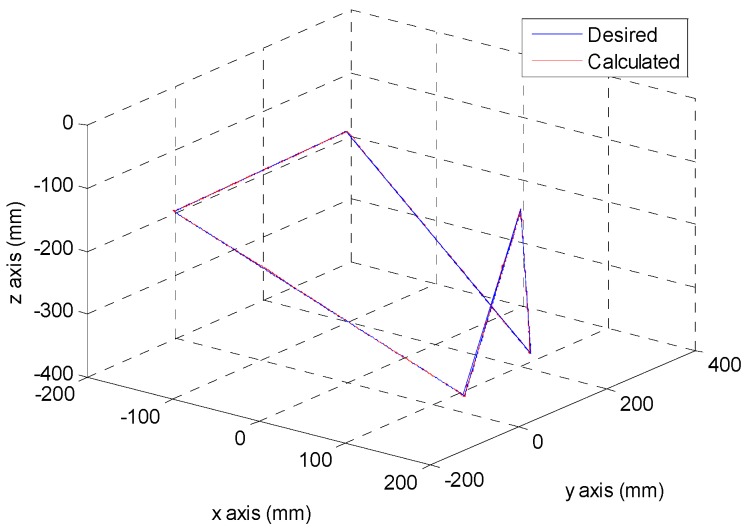
The desired and calculated trajectory.

**Figure 20:**
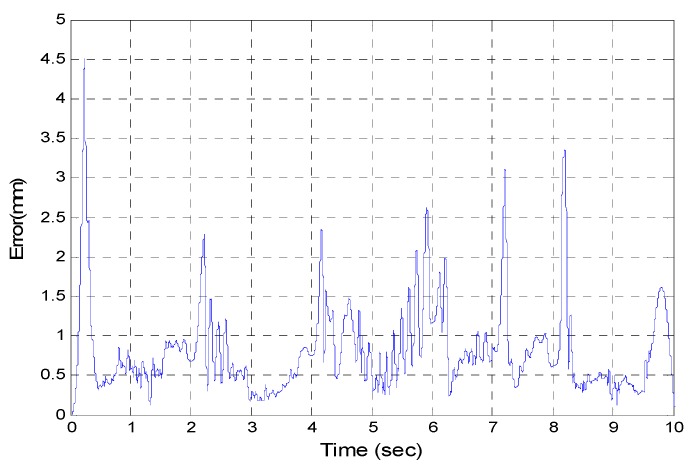
Calculated end-effector trajectory tracking error.

**Figure 21:**
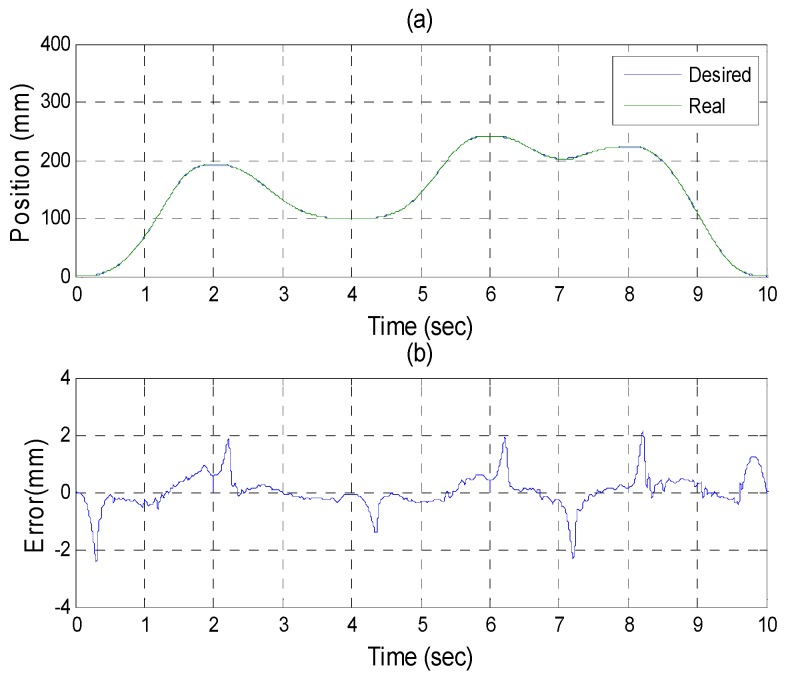
Experiments for A axis cylinder (**a**) tracking responses (**b**) tracking errors.

**Figure 22:**
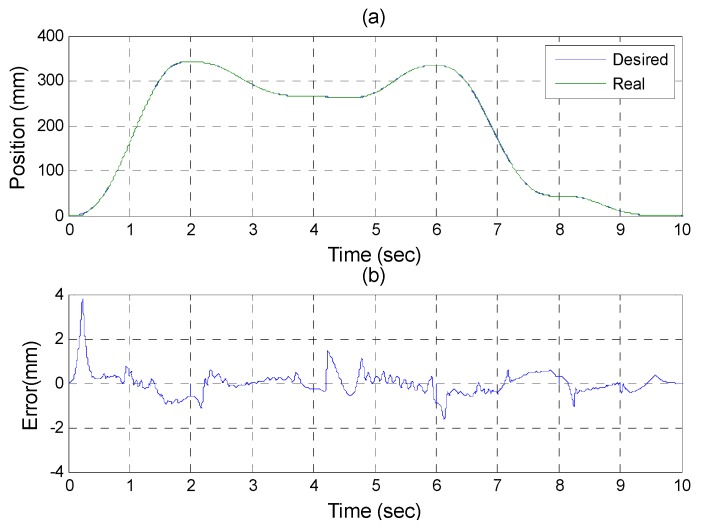
Experiments for B axis cylinder (**a**) tracking responses (**b**) tracking errors.

**Figure 23:**
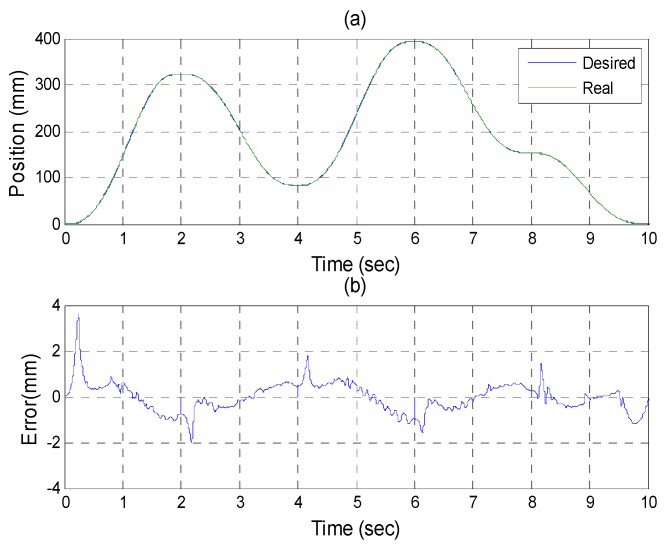
Experiments for C axis cylinder (**a**) tracking responses (**b**) tracking errors.

After the poker pattern is recognized by the SURF feature point detection with the RANSAC algorithm and the user selects the targets for grasping by counting active fingers, the depth camera will estimate the center of each targets in the end-effector frame by the coordinate transform from the camera frame. Once the pick and place locations are calculated, the program will automatically generate the customized 5th order trajectory of the end-effector for path tracking control. The experiments are from (X, Y, Z) = (−150, −100, −150) mm back to (0, 0, 0) mm in 2 s. The red line of Figure 19 illustrates the estimated trajectory of the end-effector calculated by the forward kinematics and experimental tracking responses of three actuators. Figure 20 demonstrates the trajectory tracking error of the end-effector for 3-DOF pneumatic parallel manipulator. Figure 21, Figure 22 and Figure 23 show the experimental results of each actuator’s responses, respectively.

## 7. Conclusions

In this paper, the developed SURF and HRI image algorithm is integrated with a 3-DOF pneumatic parallel manipulator so that manipulator can define objects by the feature information of the image through the SURF algorithm with scale- and rotation-invariants, and then it can automatically move to the object, grasp it, and finally move to the desired location.

In the feature matching, we match all feature correspondences by means of image plane transformation (homography) solved by RANSAC outlier rejection. Therefore, the center of object in the image coordinates can be estimated by the average of the four corners of the reference image.

Xtion Pro Live was introduced and implemented for measuring the 3-D locations of target points. Furthermore, we developed a coordinate transform calibration method for eye-to-hand calibration using the least squares and pseudo inverse methods.

The gesture recognition for counting active fingers was used to select the desired object to be grasped. When each pick and place location is confirmed in the end-effector reference frame, the program will generate the 5th order trajectories for the path tracking control.

All of the theorems in this paper are derived and verified in the experiments. The three-axial pneumatic parallel manipulator can recognize each target pattern in a workspace then pick and place it successfully.

## Figures and Tables

**Table 1:** The depth camera’s intrinsic parameters.

Depth Camera		
Focal Length (pixel)	Horizontal	*f_x_* = 577.55158
	Vertical	*F_y_* = 579.65506
Skew	*γ* = 0 (not considered)	
Principle Point (pixel)	317.47191	243.0783
Distortion (Radial)	*k*_1_ = –0.01425	*k*_2_ = 0.001
Pixel Error	*e* = [0.21365, 0.22484]	
